# TSA Suppresses miR-106b-93-25 Cluster Expression through Downregulation of MYC and Inhibits Proliferation and Induces Apoptosis in Human EMC

**DOI:** 10.1371/journal.pone.0045133

**Published:** 2012-09-19

**Authors:** Zhi-Ning Zhao, Jiu-Xu Bai, Qiang Zhou, Bo Yan, Wei-Wei Qin, Lin-Tao Jia, Yan-Ling Meng, Bo-Quan Jin, Li-Bo Yao, Tao Wang, An-Gang Yang

**Affiliations:** 1 State Key Laboratory of Cancer Biology, Department of Immunology, Fourth Military Medical University, Xi'an, Shaanxi, China; 2 Department of Biochemistry and Molecular Biology, Fourth Military Medical University, Xi'an, Shaanxi, China; 3 Department of General Dentistry and Emergency, School of Stomatology, Fourth Military Medical University, Xi'an, Shaanxi, China; 4 Department of Hematology, Tangdu Hospital, Fourth Military Medical University, Xi'an, Shaanxi, China; 5 Clinical Laboratory, 451 Hospital of Chinese People's Liberation Army, Xi'an, Shaanxi, China; 6 Department of Blood Purification, Shenyang General Hospital of People's Liberation Army, Shenyang, China; Roswell Park Cancer Institute, United States of America

## Abstract

Histone deacetylase (HDAC) inhibitors are emerging as a novel class of anti-tumor agents and have manifested the ability to decrease proliferation and increase apoptosis in different cancer cells. A significant number of genes have been identified as potential effectors responsible for the anti-tumor function of HDAC inhibitor. However, the molecular mechanisms of these HDAC inhibitors in this process remain largely undefined. In the current study, we searched for microRNAs (miRs) that were affected by HDAC inhibitor trichostatin (TSA) and investigated their effects in endometrial cancer (EMC) cells. Our data showed that TSA significantly inhibited the growth of EMC cells and induced their apoptosis. Among the miRNAs that altered in the presence of TSA, the *miR-106b-93-25* cluster, together with its host gene *MCM7*, were obviously down-regulated in EMC cells. *p21* and *BIM*, which were identified as target genes of *miR-106b-93-25* cluster, increased in TSA treated tumor cells and were responsible for cell cycle arrest and apoptosis. We further identified *MYC* as a regulator of *miR-106b-93-25* cluster and demonstrated its down-regulation in the presence of TSA resulted in the reduction of *miR-106b-93-25* cluster and up-regulation of *p21* and *BIM*. More important, we found *miR-106b-93-25* cluster was up-regulated in clinical EMC samples in association with the overexpression of *MCM7* and *MYC* and the down-regulation of *p21* and *BIM*. Thus our studies strongly indicated TSA inhibited EMC cell growth and induced cell apoptosis and cell cycle arrest at least partially through the down-regulation of the *miR-106b-93-25* cluster and up-regulation of it's target genes *p21* and *BIM* via *MYC*.

## Introduction

Endometrial cancer (EMC) is a common gynecologic malignancy, comprising approximately 20%∼30% of all female genital tract malignant tumors. In recent years, the morbidity of EMC has tended to increase, exceeding that of cervical carcinoma. Undoubtedly, studying the mechanism underlying the malignant phenotype will help to develop effective therapies for EMC [1], [2].

Recently, many studies have focused on epigenetic aberrations and their contribution to malignant transformation and progression of cancer. The malignant phenotype of EMC may be related to epigenetic modifications. The mechanisms of epigenetic control of genes involve DNA methylation and histone modifications without the alteration of the nucleotide sequence of the genes. Such modifications include acetylation, methylation, phosphorylation and ubiquitination of specific amino acid residues of the N-termini of core histones [3]. Among all of these histone modifications, histone acetylation/deacetylation plays a central role in epigenetic regulation of genes [4]. These core histones, being widely and irregularly spaced, render the gene accessible to the transcriptional apparatus. By contrast, the hypermethylation within gene promoters facilitates binding of methyl-CpG binding domain proteins (MBDs). The histone deacetylase (HDAC) in turn is recruited to the promoter region by MBDs. The histones then become deacetylated and methylated, forming tightly compacted nucleosomes inaccessible to transcription elements [5]. Deacetylated histones at the promoter regions of certain genes are detected in type I and type II EMCs, suggesting divergent epigenetic backgrounds and unique tumorigenic pathways [6]. Furthermore, expression of class I histone deacetylases indicates poor prognosis in endometrial carcinomas, and alterations of HDAC and/or HAT expression are potentially involved in impaired endometrial differentiation [7].

HDAC inhibitors are a novel class of chemotherapeutic agents, which were initially identified because they have the ability to reverse the malignant phenotype of transformed cells. HDAC inhibitors have been shown to exert various anti-tumor effects, such as growth inhibition, the induction of apoptosis or differentiation, and the blocking of angiogenesis in vivo [7], [8]. Trichostatin A (TSA), a Streptomyces product, was the first discovered natural product that inhibits the activity of Class I and II HDACs. It can inhibit cancer cell growth *in vitro* and *in vivo*, revert oncogene-transformed cell morphology, induce apoptosis, and enhance cell differentiation [9].

MicroRNAs (miRNAs) are non-coding RNAs of 22 nt that function as post-transcriptional regulators. By base-pairing with the complementary sites in the untranslated region (UTR) of mRNA, miRNAs can control the mRNA stability and the efficiency of translation [10]. Recent evidence has shown that miRNA mutations or mis-expression correlate with various human cancers, and indicating that miRNAs can function as either tumor suppressors or oncogenic RNAs [11].

Several studies have focused on the significance of miRNAs in EMC, most of them by describing the miRNA profiles of the tumor cells. Other studies have described the possible role of histone acetylation in the development and progression of EMC. Because of the profound effects of HDAC inhibitors on gene expression, it is certain that they have the potential to regulate miRNA levels [12]. Nevertheless, few reports have attempted to explain how HDAC regulates the malignant progress of EMC via miRNAs. Recently, our group used microarray technology to determine the differentially expressed miRNAs in EMC by treating ECC-1 cells with tamoxifen (TAM) alone or together with TSA. The *miR-106b-93-25* cluster was found to be decreased significantly in cells treated with TAM and TSA, but not in cells treated with TAM alone. This result suggested strongly that TSA plays a regulatory role in the expression of this miRNA cluster.

The *miR-106b-93-25* cluster consists of three miRNAs, *miR-106b*, *miR-93* and *miR-25*, and is located in the 13th intron of the minichromosome maintenance protein 7 (*MCM7*) gene of human chromosome 7. This miRNA cluster is upregulated in many human cancers, such as gastric, prostate, and pancreatic neuroendocrine tumors, The miRNAs of the *miR-106b-93-25* cluster are co-transcribed in the context of the *MCM7* primary transcript. Furthermore, *MCM7* overexpression is an indicator of poor prognosis in EMC. These observations raise the possibility that *MCM7* oncogenic properties can be linked, at least in part, to the hosted miRNAs.

The MYC protein family is comprised of basic helix-loop-helix-zipper (bHLHZ) transcription factors (c-, N-, and L-MYC) that can each form obligate heterodimers with the small bHLHZ protein Max. MYC, as a transactivator, binds to a core E-box promoter element CAC/TGTG after forming a heterodimer with Max. The *MCM7* promoter has an E-box binding site for the MYC oncoproteins. Deregulated expression of MYC has been implicated in the genesis of many types of tumors [13].

These findings prompted us to determine whether MYC might contribute to endometrial oncogenesis through regulation of miRNAs and the effects and mechanism of TSA on EMC cells.

## Materials and Methods

### Plasmids

In order to generate an gene/luciferase reporter plasmid, the 3′ UTRs from the human p21CIP1 (p21 herein after) and BIM genes were cloned into a vector containing the luciferase open reading frame pGL3-control-MCS2 reporter vector which was reconstructed by Wang Tao (Department of Immunology, Basic Medical College, Forth Military Medical University, Xi'an, China). We also constructed plasmids containing the p21-3′UTR with mutated seed regions for the predicted miR-106b/miR-93 binding sites (p21-mut-3′UTR), along with plasmids containing the BIM-3′UTR with mutated seed regions for the predicted miR-25 binding sites (BIM-mut-3′UTR). Primer sequences are available in Table S1.

An 800-bp MCM7 promoter construct, corresponding to the sequence from −756 to +44 (relative to the TSS) of the 5′-flanking region of the human MCM7 gene, was generated from human genomic DNA using forward and reverse primers. Using the (−756/+44) MCM7 construct as a template, several deletion constructs of the MCM7 promoter, including −570/+44, −500/+44, −403/+44, −185/+44, −70/+44 and −52/+44 were similarly generated by corresponding forward primers. The obtained constructs were confirmed by DNA sequencing and cloned into the pGL3 Basic vector (Promega, Madison, WI) carrying the luciferase reporter gene, to obtain the pGL3-MCM7LUC plasmid. Point mutations in the E-box site, were generated in the pGL3-185/+44 construct using standard site-directed mutagenesis procedures. A mutant reverse primer 5′-GCCCCCgaCGTGACCGGCGCCACTGCG-3′ was annealed in combination with the previously described forward primer and used for PCR amplification. Meanwhile, a mutant forward primer 5′- gccggtcacgGAgggggcgacgtttcgcgc-3′ was annealed in combination with the previously described reverse primer and used for PCR amplification. The amplified product was gel purified and ligated into the pGL3-Basic vector.

The pBABE-MYC construct was generously provided by Zhang Rui (Department of Biochemistry and Molecular Biology, Basic Medical College, Fourth Military Medical University, Xi'an, China).

Primer sequences are available in Table S1.

### Cell culture and TSA treatment

The EMC cell lines, including ECC-1 and HEC-1A cell lines, were obtained as a kind of gift from Shang Yongfeng [14] (Department of Biochemistry and Molecular Biology, Basic Medical College, Peking University, Beijing, China) and purchased from the Cell Bank of the Chinese Academy of Sciences(Shanghai, China) by Li Yan (Department of Biochemistry and Molecular Biology, Basic Medical College, Fourth Military Medical University, Xi'an, China), respectively. Cells were maintained in 25-cm2 flasks (Costar, Cambridge, MA), with RPMI-1640 (GIBCO, Carlsbad, CA) for ECC-1 cells and DMEM (GIBCO, Carlsbad, CA) for HEC-1A cells supplemented with 10% fetal bovine serum (FBS, Invitrogen, Carlesbad, CA) at 37°C in the presence of 5% CO2. To test the expression of internal standards, cells with fresh media were treated with either a vehicle (ethanol) or TSA(Sigma,St.Louis, MO) at the concentrations of 100 ng/mL for 24 h.

### miRNA mimics, inhibitors and plasmids transfection

Cells were cultured to 60% confluency in RPMI 1640 (ECC-1) or DMEM (HEC-1A). Synthesized RNA duplexes of miRNA mimics, miRNA inhibitors, and siRNA duplex against MYC were purchased from Shanghai GenePharma (Shanghai, China). Cells were transfected with 50 nM of each miRNA mimic and inhibitor or siRNA as indicated. At 6 h post-transfection, the medium was changed to complete medium.

For siRNA-mediated target knockdown, three siRNAs (GenePharma, Shanghai, China) were transfected at 50 nM each or pooled at 16 nM of each duplex. The control siRNA used in all experiments targets the firefly luciferase gene, which is not present in these cells.

The MYC expression vector (pBABE-MYC) was transfected with Lipofectamine 2000 (Invitrogen) in ECC-1 cells at the concentration 50 nM. For TSA (Sigma) incorporation analysis, 48 h after transfection, ECC-1 cells were pulsed with TSA for 24 h (Sigma).

Sequences for siRNA oligo-duplexes and RNA duplexes corresponding to mature microRNAs are listed in the Table S1.

### Cell proliferation assay

ECC-1 cells (1,000 per well) transfected with synthetic miRNAs or treated with TSA were grown in 96-well plates. Following the manufacturer's protocol, cell proliferation was documented every 24 h by adding 10 µL of MTT (3-(4,5-dimethylthiazol-2-yl)-2,5-diphenyltetrazo-lium bromide, Roche Molecular Biochemicals) to each well incubating at 37°C for 4 h, followed by the addition of 100 µL DMSO in each well. Absorbance was measured at 490 nm in an ELISA reader (Microplate reader model 550, USA).

### Cell cycle analysis

Cells from each well were harvested by trypsinization, and then fixed with 95% ethanol at 20°C for 1 h. Cell cycle distributions were measured by staining with propidium iodide as described previously [32], followed by analysis on a FACS Calibur flow cytometer (Becton Dickinson, Bioscience, USA). A total of 10,000 events were counted for each sample. In each instance, flow cytometry was performed at least twice, and a representative experiment is shown in each figure.

### Flow cytometry

The Annexin V-FITC kit (BD Biosciences, San Diego, CA) was used to label apoptotic cells. Cells transfected with miRNAs or treated with TSA were washed with cold PBS and diluted in 1× Annexin binding buffer at a concentration of 1×106 cells/ml. The cells (1×105) were mixed with 5 µL of Annexin V-FITC stock solution and the binding carried out at room temperature for 15 min in the dark. The samples were diluted to 400 µL and immediately analyzed by flow cytometry for apoptotic cells.

### RNA isolation and qRT-PCR analysis

Treated cells were lysed in Trizol reagent (Invitrogen), and total RNA was extracted according to the manufacturer's instructions. RNA was quantified using a spectrophotometer (Beckman Coulter, USA). For miRNA analysis, the total RNA isolated was diluted to a 100 ng/µL working dilution and used in the reverse transcriptase reaction. Analysis of miRNAs was performed using the miScript system (Qiagen, Valencia, CA), consisting of the miScript Reverse Transcription kit, miScript Primer assays and miScript SYBR Green PCR kit, according to the protocol provided by the company. Small nuclear RNA U6 was used for normalization. For mRNA analysis, RNA was isolated as mentioned above, diluted to a 100 ng/µL stock reverse transcribed using the M-MLV Reverse transcriptase (Invitrogen). qRT-PCR was performed using SYBR Premix Ex TaqTM II (TaKaRa) on an ABI 7901HT series PCR machine Applied Biosystems, and data were normalized to GAPDH expression and further normalized to the negative control. All of the PCR reactions were performed in triplicate and independently repeated at least two to three times. The primers used in PCR amplification are listed in the Table S1.

### Western blot assay

Cells were washed twice with PBS, then ice-cold lysis buffer (25 mM Tris-HCl, pH 7.5, 150 mM NaCl, 1 mM EDTA, 1% Triton X-100 and a mixture of proteases inhibitors) was added. The cells were rapidly scraped off the plates, and the crude lysates were transferred to pre-chilled Eppendorf tubes and centrifuged at 12,000× g for 15 min at 4°C. The protein concentrations were quantified using the Bio-Rad protein Assay Reagent (Bio-Rad, CA) according to the manufacturer's protocol. The whole cell lysates (50 µg) were resolved on a 12% SDS-polyacrylamide gel, transferred to a nitrocellulose membranes and probed sequentially with antibodies from Santa Cruz Biotechnology (Santa Cruz, CA) against p21, BIM and MYC or against an antibody against β-actin from Sigma. The blots were developed using the Multilmage light cabinet (Alpha Innotech, USA).

### Reporter gene assays

Transcriptional inhibition of the luciferase reporter gene by either miR-106b or miR-25 was assayed in ECC-1 cells. Briefly, 1.0×105 ECC-1 cells per well were seeded in 24-well plates. The next day, cells were transfected with 100 ng of pGL3-p21-3′UTR and pGL3-BIM-3′UTR or their mutant counterparts, 5 ng pRL-TK Renilla plasmid (Promega) and either miRNA negative control or miRNA mimics at a final concentration of 100 nmol/L using the Lipofectamine 2000. For detecting the activity of MYC on the human MCM7 promoter assay, a series of expression plasmids of the MCM7 promoter (100 ng) were co-transfected with 5 ng pRL-TK Renilla plasmid (Promega) and 100 ng pBABE-MYC using Lipofectamine 2000. Twenty-four hours after transfection, the luciferase activity was measured by using the dual-luciferase reporter assay system (Promega). Each experiment was performed in triplicate.

### ChIP assay

Approximately 2×10^7^ ECC-1 cells were collected. ChIP was performed using a Human MYC Chromatin Immunoprecipitation Kit (Millipore Corporation, MA). Cells were incubated for 15 min in medium containing 1% formaldehyde at room temperature, and cross-linking was quenched by adding glycine to 125 mM. The solution was swirled for 5 minutes at room temperature, and then the cells were pelleted. After removing the media, the cell pellet was resuspended in 500 µL of Lysis Buffer containing a protease inhibitor cocktail (Sigma) and incubated on ice for 10 min. Sonication was performed by pulsing three times for 15 s, incubating on ice for 2 min between each pulse, followed by centrifugation at 12,000× *g* for 5 min to remove cell debris. Analysis by gel electrophoresis indicated that a substantial fraction of the DNA fragments at this stage were ∼1.0 kb in length. The lysates were centrifuged for 10 min using a refrigerated ultracentrifuge at 12,000× g. The Supernatants were collected, diluted by adding 1 mL of Dilution Buffer and then centrifuged again for another 15 min to obtain cleared cell lysate. ChIP was performed on the cell lysate by overnight incubation on a rotating device at 2–8°C with 5 µL of anti-MYC antibody or 20 µL of normal goat IgG as a negative control. Streptavidin magnetic beads (50 µL) were added to the samples and rotated for 30 min at 4°C. The tube was applied to a magnet for 2 min to collect the beads. After rinsing four times sequentially with wash buffer, the beads were then bioled for 10 min with 100 µL Chelating Resin Solution. DNA samples were then purified using a DNA purification kit. One microliter of DNA sample was used in PCR reactions. Primer sequences used in this assay are listed in the Table S1.

### Tissue specimens

All human specimens were encoded to protect patient confidentiality and processed under protocols approved by the Xijing Hospital Ethics Committee. Tumors and the corresponding normal tissues were obtained from patients with primary EMC at the Xijing hospital (the first affiliated hospital of the Fourth Military Medical University, Xi'an, China). After surgical removal, the tissues were frozen immediately in liquid nitrogen and stored at −80°C. Written informed consent for the studies was obtained from all patients.

### Statistical analysis

In order to evaluate significant differences, t tests and analyses of variance (ANOVA) on independent samples were performed using the statistical software SPSS (version 10.0; SPSS, Chicago, IL). The statistical significance was set at ***, P<0.001; **, P<0.01; *, P<0.05.

## Results

### TSA inhibits EMC cells growth and downregulated *miR-106b-93-25* cluster and its host gene *MCM7*

**Figure 1:**
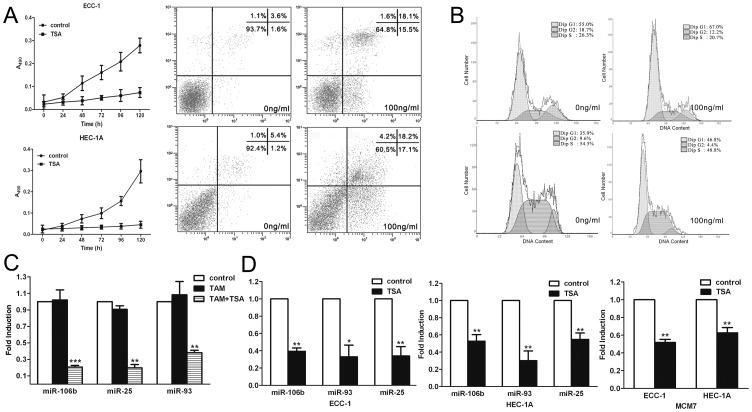
TSA inhibited EMC cells proliferation and downregulated miR-106b-93-25 cluster and its host gene MCM7. (**a**) Growth inhibitory effects of TSA on ECC-1 cells and HEC-1A cells (left) and Cytometric detection of apoptosis in cells treated with TSA (right) by Annexin V-FITC staining of EMC cells, ECC-1 cells (top) and HEC-1A cells (bottom). (**b**)Cell cycle analysis of ECC-1 (top) and HEC-1A cells (bottom) by flow cytometry. Control cells were treated with vehicle alone. Results represent the mean ± SD of three independent experiments. (**c**)Microarray results showing decreased expression levels of miR-106b, miR-93 and miR-25 in the ECC-1 cells treated with TAM (5 nM) combined with TSA (100 ng/mL) while they were unchanged when treated with TAM alone compared with control. (**d**) qRT-PCR analysis of miR-106b, miR-93, miR-25 and their host gene MCM7 expression in ECC-1 and HEC-1A cells treated with TSA (100 ng/mL) for 24 h. Relative expression levels were normalized using the human housekeeping GAPDH gene. Error bars represent the S.D. of mean values (expressed as percent of control) from three independent experiments. ***, P<0.001; **, P<0.01; *, P<0.05, paired t-test.

We examined the effects of the HDAC inhibitor TSA on the growth of both ECC-1 and HEC-1A EMC cell lines *in vitro*. TSA (100 ng/mL) exerted strong growth inhibition of both cell lines (Fig. 1). To determine if the cell death observed was due to induced apoptosis after TSA administration, the cells were analyzed using flow cytometry. Following treatment with TSA (100 ng/mL) for 24 h, the cells were stained with Annexin V, a phospholipid binding protein that specifically recognizes phosphatidylserine exposed on the cell surface and indicates apoptosis (Fig. 1A). The proportion of apoptotic nuclei increased up to 35% in ECC-1 or 39% in HEC-1A cells after treatment with TSA. The results indicated that a significantly increased number of cells died following TSA treatment, confirming the potency of this reagent in activating cell death pathways. The relative proportions of cells undergoing apoptosis following TSA treatment was consistent with the sensitivity profiles established by cell growth curves (Fig. 1A). The effect of TSA on cell cycle progression was next examined. ECC-1 and HEC-1A cells cultured with TSA (100 ng/mL) for 24 h showed an accumulation of cells in the G1-G0 phases of the cell cycle, with a concomitant decrease in the proportion of those in S phase, *e.g.*, a total of 55% of the untreated ECC-1 cells was in G1 phase compared with 67% of cells cultured with TSA; 36% of the untreated HEC-1A cells was in G1 phase compared with 47% of cells cultured with TSA (Fig. 1B).

In previous research from our lab, we performed microarray analysis to identify differentially expressed miRNAs in ECC-1 cells treated with TAM (0.5 µM) or TAM combined with TSA (100 ng/mL). We found the expressions of *miR-106b*, *miR-93* and *miR-25* were decreased in the group treated with TAM and TSA, while they were unchanged in the group treated with TAM only, compared with the control (Fig. 1C). This result suggested that TSA may regulate the expressions of *miR-106b*, *miR-93*, and *miR-25*. In order to verify the alteration in expression level of this cluster, we used quantitative real-time PCR (qRT-PCR) to detect these three miRNAs in total RNA isolated from cultured ECC-1 and HEC-1A cells with or without TSA (100 ng/mL) for 24 h. The expressions of miR-106b, miR-93, and miR-25 were shown to be downregulated significantly in cells treated with TSA compared to control (Fig. 1D & C), consistent with our microarray results (Fig. 1C).

These three miRNAs clustered (*miR-106b-93-25*) within intron 13 of *MCM7* are actively co-transcribed with the *MCM7* primary RNA transcript [15]. *MCM7* plays a pivotal role in the G1/S phase transition, orchestrating the correct assembly of replication forks on chromosomal DNA and ensuring that the whole genome is replicated only once during each cell cycle [16]. As overexpression of *MCM7* has been associated with poor prognosis in prostate cancer and EMC [17], we hypothesized that the oncogenicity of *MCM7* may be linked, at least in part, to overexpression of the hosted miRNAs. Thus, we hypothesized that activation of these miRNAs should correlate with activation of their host gene *MCM7*. To investigate this hypothesis, we examined *MCM7* mRNA levels in the same cell lines using qRT-PCR. Indeed, we found that *MCM7* mRNA expression was also downregulated in cells cultured with TSA (Fig. 1D).

### The *miR-106b-93-25* cluster promotes endometrial cell growth and inhibits cell apoptosis

**Figure 2:**
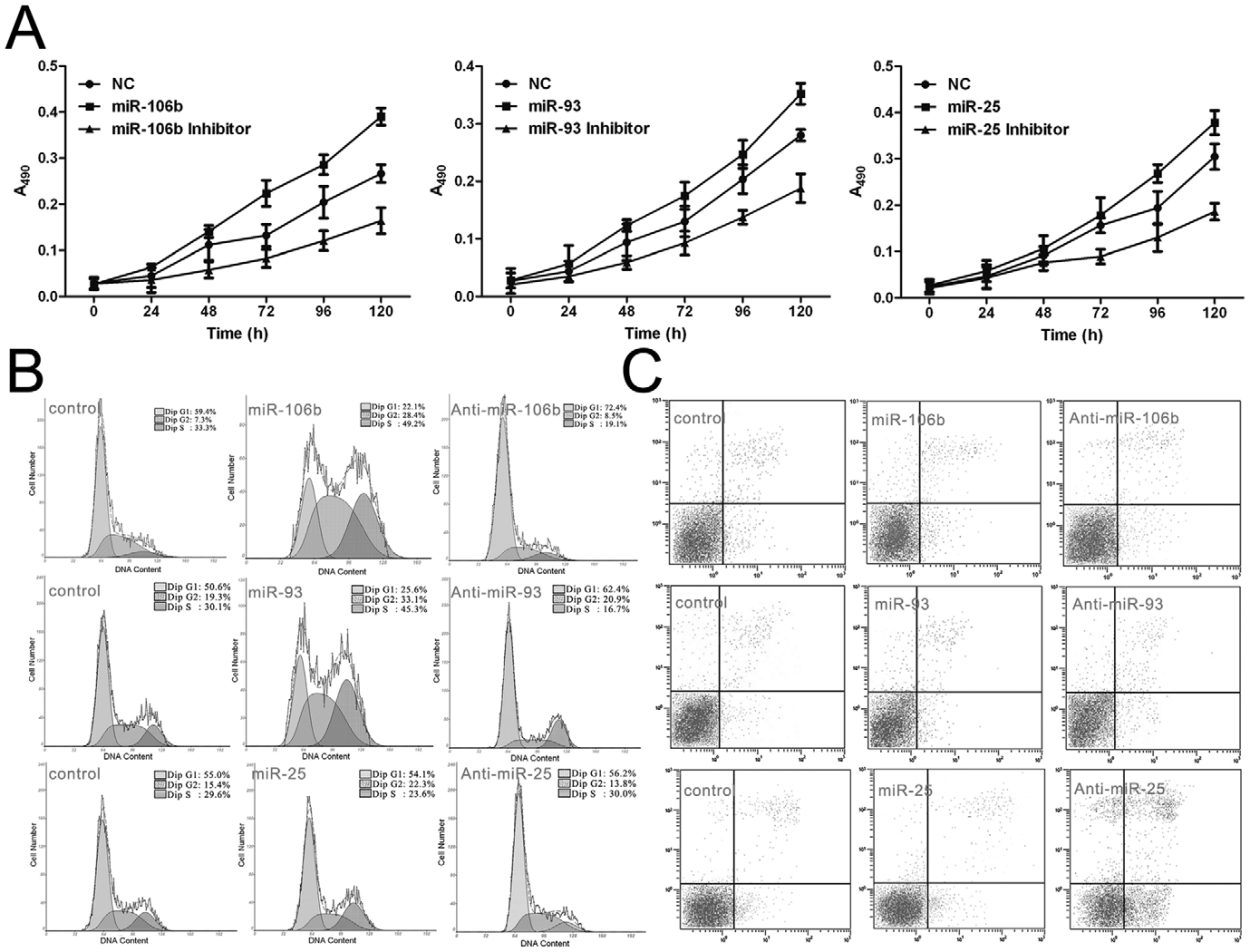
The *miR-106b-93-25* cluster promoted EMC cell growth. (**a**)At indicated times post-transfection, MTT assays were used to compare the effects of *miR-106b*, *miR-93*, *miR-25* and their inhibitors. *miR-106b*, *miR-93*, *miR-25* promoted the growth of ECC-1 cells, while their inhibitors significantly decreased proliferation in ECC-1 cells. Values represent the means ± SD from triplicate determinations. (**b**) Effects of *miR-106b*, *miR-93* or *miR-25* on the cell cycle. FACS analysis was performed on cells transfected with either miRNA or miRNA inhibitors. *miR-106b* and *miR-93* gain of function led to an increase in cells in S-phase, while the G1-phase population was increased in the cells transfected with their inhibitors. miR-25 gain of function had no effects on the cell cycle. (**c**) Effects of *miR-106b* or *miR-93* or *miR-25* on apoptosis. FACS analysis was performed on cells transfected with either miRNA or miRNA inhibitors. miR-25 gain of function decreased apoptosis of cells. Apoptosis increased significantly in cells transfected with the *miR-25* inhibitor. *miR-106b* and *miR-93* had no effect on the apoptosis levels of the cells.

To study the effects of the *miR-106b-93-25* cluster on cell proliferation and cell cycle progression, we transfected synthetic RNA duplexes (to mimic the miRNAs) or anti-miRs (to inhibit the miRNAs) into ECC-1 cells. MTT assays and flow cytometry were used to detect the effects of the *miR-106b-93-25* cluster on EMC cells. The *miR-106b*, *miR-93* and *miR-25* duplexes promoted cell proliferation compared with a control duplex, whereas their inhibitors significantly decreased proliferation of ECC-1 cells (Fig. 2A). The cell cycle profile was then analyzed by flow cytometry. The proportion of cells transfected with *miR-106b*, or *miR-93* in the G1 phase fell, while that of cells transfected with *miR-25* remained unchanged (Fig. 2B). We also analyzed the effect of anti-miRNA oligonucleotides on the cell cycle. Cell numbers in G1 phase were reproducibly increased by anti-*miR-106b* and anti-*miR-93* but were unchanged by anti-*miR-25* (Fig. 2B). When we examined the ECC-1 cells treated with TSA by flow cytometry, gain of *miR-25* function was shown to decrease the apoptosis level in the cells. Meanwhile, the apoptosis level increased significantly in the cells transfected with the *miR-25* inhibitor. However, *miR-106b* and *miR-93* had no appreciable apoptotic effects on the cells (Fig. 2C), suggesting that these miRNAs from the cluster had more subtle effects.

### The *miR-106b-93-25* cluster downregulates the expression of *p21* and *BIM*

**Figure 3:**
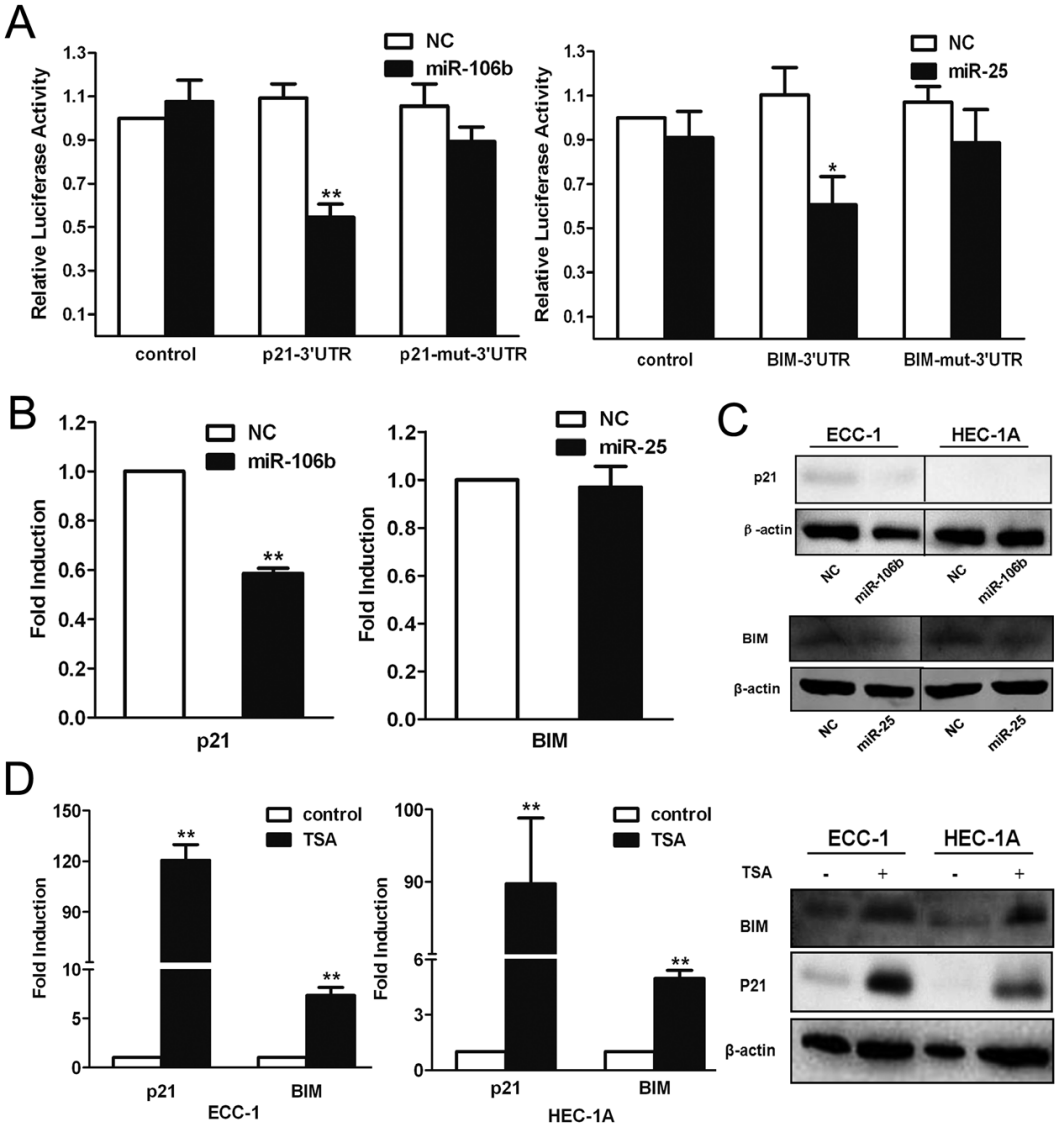
Regulation of P21 and BIM by the miR-106b-93-25 cluster. (**a**) pGL3 luciferase reporter constructs containing either the wild-type or mutant 3′UTR target sequence of miR-106b or miR-25 in the P21 or BIM gene were co-transfected into ECC-1 cells with either miRNA-negative control, miRNA mimics or empty pGL3 control vector (each n = 3). Luciferase activity was determined in the cell extracts after 24 h. In the presence of the wild-type P21 3′UTR, the miR-106b mimics significantly inhibited the luciferase activity compared with vector control. This inhibition was not observed with the mutant 3′UTR reporter construct (left). In the presence of the wild-type BCL2L11 3′UTR, the miR-25 significantly inhibited the luciferase activity compared with vector control. This inhibition was attenuated with the mutant 3′UTR reporter construct (right). (**b**) qRT-PCR analysis of P21 and BIM mRNA levels after the transfection of ECC-1 cells with miR-106b or miR-25 mimics. P21 mRNA was significantly decreased by miR-106b mimics while BIM mRNA was not significantly changed by miR-25 mimics. (**c**) Western blot analysis showing downregulated P21 and BIM proteins in ECC-1 and HEC-1A cells transfected with miR-106b and miR-25 mimics. (**d**) qRT-PCR and Western blot analysis showing upregulated P21 and BIM mRNA and protein level in ECC-1 and HEC-1A cells treated with TSA for 24 h. **, P<0.01; *, P<0.05, paired t-test.

From the results above, we presumed that TSA treatment repressed the expression of *miR-106b-93-25* cluster and promoted the expression of its targets, thereby inhibiting cells proliferation and inducing cell cycle arrest. According to previous reports, *p21* and *BIM* are the direct targets and inhibited by *miR-106b* and *miR-25*, respectively [18]. To corroborate these findings in EMC cells, a dual-luciferase reporter system was used to detect inhibition of *p21* and *BIM* expression by *miR-106b* and *miR-25* in ECC-1 cells, respectively. miRNA-negative control (NC) or miRNA mimics and a reporter vector containing either the wild-type or mutant 3′UTR of the two genes, *p21* and *BIM*, were co-transfected into ECC-1 cells. After 24 h, luciferase activity was determined in the cell extracts (Fig. 3). The mutant 3′UTRs contained a point mutation in the *miR-106b* and *miR-25* seed region complementary sites. Co-transfection of either *miR-106b* with the reporter construct containing the wild-type 3′UTR of *p21* or *miR-25* with the reporter construct containing the wild-type 3′UTR of *BIM* resulted in a significant inhibition of the luciferase reporters when compared with the miRNA negative control. There was no inhibition of the reporter by the miRNAs in the absence of the 3′UTR. The presence of a mutant 3′UTR either abolished or attenuated the effect of the miRNAs (Fig. 3A). Thus, *p21* was directly regulated by *miR-106b* through the 3′UTR, while *BIM* was directly regulated by *miR-25*. We also measured the mRNA and protein levels of p21 and BIM after *miR-106b* and *miR-25* overexpression. The *p21* mRNA level was reduced by the *miR-106b* duplex, and the same change was detected at the protein level (Fig. 3B). This result suggested mRNA decay is one of the mechanisms of *p21* inhibition by miRNA. However, the mRNA level of *BIM* was only slightly reduced by *miR-25* treatment (Fig. 3B), while its protein level was reduced significantly(Fig. 3C), indicating that repression of BIM may be mainly due to translational inhibition. Next, we detected the mRNA and protein expression levels of p21 and BIM in ECC-1 cells treated with TSA. The qRT-PCR results demonstrated that the mRNA expression levels of *p21* and *BIM* were upregulated significantly in ECC-1 and HEC-1A cells treated with TSA, while the corresponding protein levels were also upregulated as confirmed by Western blot (Fig. 3D).

### TSA suppresses *miR-106b-93-25* cluster expression through downregulation of MYC

**Figure 4:**
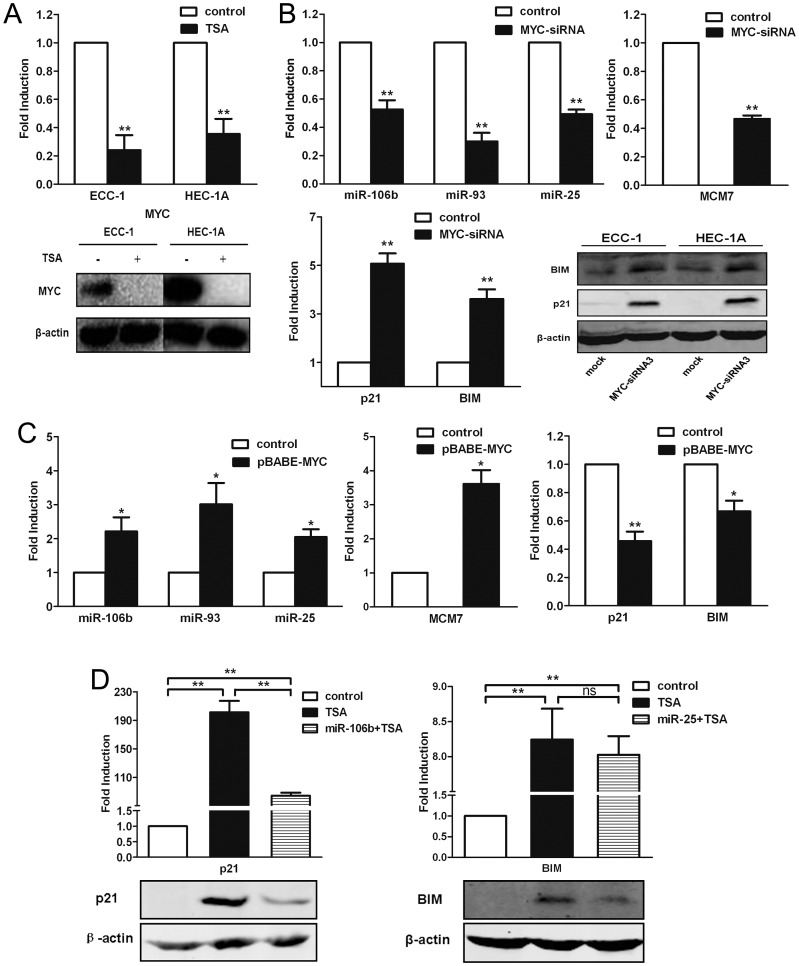
TSA downregulated the miR-106b-93-25 cluster by downregulating MYC. (**a**) The mRNA and protein levels of MYC was downregulated in ECC-1 and HEC-1A cells treated with TSA for 24 h. (**b**) Depletion of MYC inhibited the expression of miR-106-93-25, as well as its host gene MCM7, and promoted the expression of P21 and BIM mRNA and protein expressions in ECC-1 and HEC-1A cells. (**c**) Overexpression of MYC upregulated expressions of miR-106b-93-25 and MCM7, and downregulated P21 and BIM mRNA expressions. (**d**) P21 mRNA and protein levels were upregulated in ECC-1 cells treated with TSA for 24 h, while it was partially neutralized by miR-106b (left). BIM mRNA was upregulated in ECC-1 cells treated with TSA for 24 h, while it was not neutralized by miR-25, but its protein level was neutralized in miR-25 overexpressing ECC-1 cells. **, P<0.01; *, P<0.05; ns, not significant; paired t-test.

According to previous reports, TSA treatment could downregulate the transcript and protein level of MYC [19]. By bioinformatic analysis, we found potential binding sites for MYC in the upstream promoter region of the *MCM7* gene. Therefore, we considered that MYC may regulate the expression of the *miR-106b-93-25* cluster as well as its host gene *MCM7*. To verify that MYC plays a role in cell apoptosis and cell cycle arrest induced by TSA treatment, we first detected the expression of MYC in ECC-1 and HEC-1A cells treated with TSA (100 ng/mL). The mRNA as well as protein expression levels of MYC were downregulated significantly in the ECC-1 and HEC-1A cells cultured with TSA (100 ng/mL) (Fig. 4A). In order to establish the importance of MYC as a regulator in this course, we suppressed MYC protein in ECC-1 cells by small interfering RNA (siRNA). ECC-1 cells were transiently transfected with three siRNA vectors targeting different sequences of human *MYC* among which siRNA3 was found to be most efficient as determined by Western blot analysis (Fig. S1). Thus, *MYC*-siRNA3 was selected for the subsequent experiments. The expression of the *miR-106b-93-25* cluster and its host gene *MCM7* were downregulated after siRNA silencing of *MYC* (Fig. 4B) and upregulated in cells overexpressing *MYC* (Fig. 4C), indicating they may be regulated by MYC. Next, we examined the change in levels of the target genes of the *miR-106b-93-25* cluster. The results of the qRT-PCR and Western blot analyses demonstrated that the mRNA and protein levels of p21 and BIM were upregulated significantly after the depletion of *MYC* (Fig. 4B). By contrast, the mRNA levels of *p21* and *BIM* were downregulated significantly in ECC-1 cells overexpressing *MYC* (Fig. 4C).

According to previous reports, TSA can upregulate the expression of *p21* via acetylating its promoter (p53-independent pathway) [20], [21] or via upregulating the expression of p53 (p53-dependent pathway) [22]. To certify the role of the *miR-106b-93-25* cluster in the upregulation of *p21* and *BIM* by TSA, the ECC-1 cells transfected with *miR-106b* or *miR-25* mimics were cultured with or without TSA for 24 h, and then the mRNA and protein expression of p21 and BIM were analyzed by qRT-PCR and Western blot assays, respectively. The mRNA and protein levels of p21 were upregulated significantly when treated with TSA alone, but the magnitudes of the increases were attenuated significantly in the cells overexpressing *miR-106b* (Fig. 4D), indicating that the induction of p21 by TSA was regulated, at least in part, by downregulation of the *miR-106b-93-25* cluster. Furthermore, similar changes were observed in the protein levels of BIM, the upregulation of protein level of BIM by TSA treatment was partially neutralized by *miR-25*. But the mRNA levels of *BIM* by TSA treatment were slightly changed in the cells overexpressing *miR-25* or not(Fig. 4D). Consistent with this, our data showed that the upregulation of p21 and BIM by depletion of *MYC* could be partially reversed by *miR-106b* and *miR-25*, suggesting that miRNA involvement is one of the reasons for the upregulation of p21 and BIM by *MYC*-siRNA. Taken together, our data strongly suggests that the *miR-106b-93-25* cluster's involvement in upregulation of p21 and BIM, different from the previously described p53-dependent and independent ways, is a novel mechanism of TSA induced EMC cell cycle arrest and apoptosis.

### MYC enhances the promoter activity of human *MCM7*

**Figure 5:**
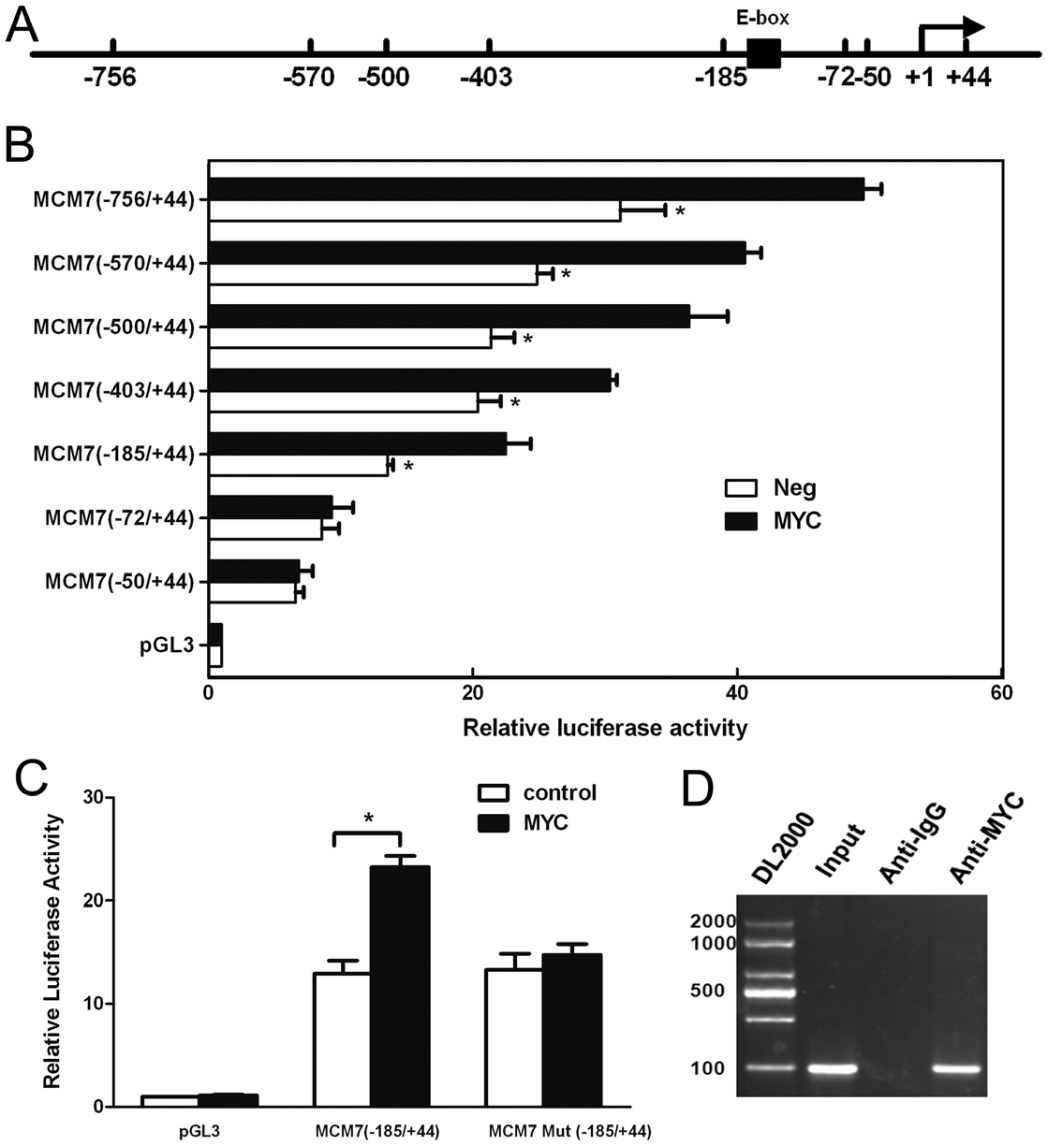
MYC activation of human *MCM7* promoter activity was dependent on the E-box sequence. ECC-1 cells were transfected to detect the human *MCM7* promoter activity. (**a**) Schematic of the core promoter region of the *MCM7* promoter. Location of the mutated sequence for the E box (AGTTTA) is indicated by the black box. (**b**) Luciferase activity of constructs of the *MCM7* promoter and its truncations. ECC-1 cells transfected with luciferase reporter constructs containing different 5′flanking segments of hMCM7 alone or co-transfected with pBABE-MYC. The numbers on the left side of the panel represent the relative position of each deletion. The luciferase activities were normalized to that of ECC-1 cells transfected with the pGL3(-) vector (control) was set to 1.0. Results are means ± S.D. All assays were repeated for at least 3 independent experiments. In each experiment, the individual data points were calculated as the means of triplicates. (**c**) Effect of mutated E-box site on activity of the *MCM7* promoter. The MCM7 (−185/+44)/luciferase reporter construct was co-transfection with its E-box site mutant and with or without the MYC expression plasmid. (**d**) ChIP assays were performed in ECC-1 cells using antibodies directed against MYC protein or an IgG control. The sequences of all primers for ChIP-PCR were based on the MYC-binding sites (−756 to −50 relative to the TSS) identified from TSS analysis. After immunoprecipitation, DNA was eluted and amplified by PCR using primers designed to amplify the minimal promoter region of MCM7. *, *P*<0.05, paired t-test.

MYC is known to bind to the E box sequence (CACGTG) by forming heterodimers with Max [23], [24] to activate transcription. As mentioned above, we had found putative MYC binding sites upstream of the *MCM7* gene promoter region by bioinformatics analysis, suggesting that MYC, via interaction at these sites, could achieve the transcriptional regulation of both *MCM7* and the *miR-106b-93-25* cluster. In order to verify the mechanism of MYC-induced transcriptional activation, we amplified the putative promoter region of the *MCM7* gene (−756/+44) (Fig. 5A). A series of deletions generating segments of different lengths of the *MCM7* promoter were subcloned into a luciferase reporter plasmid and used to confirm the MYC binding site. These plasmids were transiently transfected alone or co-transfected with pBABE-MYC into ECC-1 cells. MYC was shown to promote the luciferase activities of pGL3-756/+44, pGL3-570/+44, pGL3-500/+44, pGL3-403/+44 and pGL3-185/+44, while no significant elevation in luciferase activities were detected with pGL3-72/+44 and pGL3-52/+44 compared to the control in ECC-1 cells (Fig. 5B). Therefore, segments of the promoter region truncated at the 5′ end from −756 up to −185 bp upstream of the transcriptional start site (TSS) produced quite high luciferase activities, while no elevated luciferase activity was observed with deletions beyond −72. These data demonstrated the strong transcriptional activity of MYC on human *MCM7*. Furthermore, the segments showing significant promoter activity (truncated from −756 to −185 relative to the TSS) contained the E-box sequence located between −135 to −126, suggesting that it is essential for MYC-mediated activation of *MCM7* promoter activity. To test this notion, a construct similar to pGL3-185/+44 but harboring a mutated E-box site was constructed and co-transfected with MYC into ECC-1 cells. This construct showed only the control level of luciferase activity (Fig. 5C).

To determine whether MYC regulates the transcriptional activity of *MCM7* by directly binding to its promoter in ECC-1 cells, we performed a chromatin immunoprecipitation (ChIP) assay with primers shown in Table S1. Binding of MYC protein to the E-box motif in the *MCM7* promoter was determined by analyzing the anti-MYC immunoprecipitated chromatin sample by PCR using a primer pair specific for the MYC-binding region of the *MCM7* promoter or a control region far downstream. The results showed that the endogenous MYC protein was recruited to the E-box sequence in the *MCM7* promoter in ECC-1 cells (Fig. 5D).

### The *miR-106b-93-25* cluster and *MYC* are overexpressed in clinical samples

**Figure 6:**
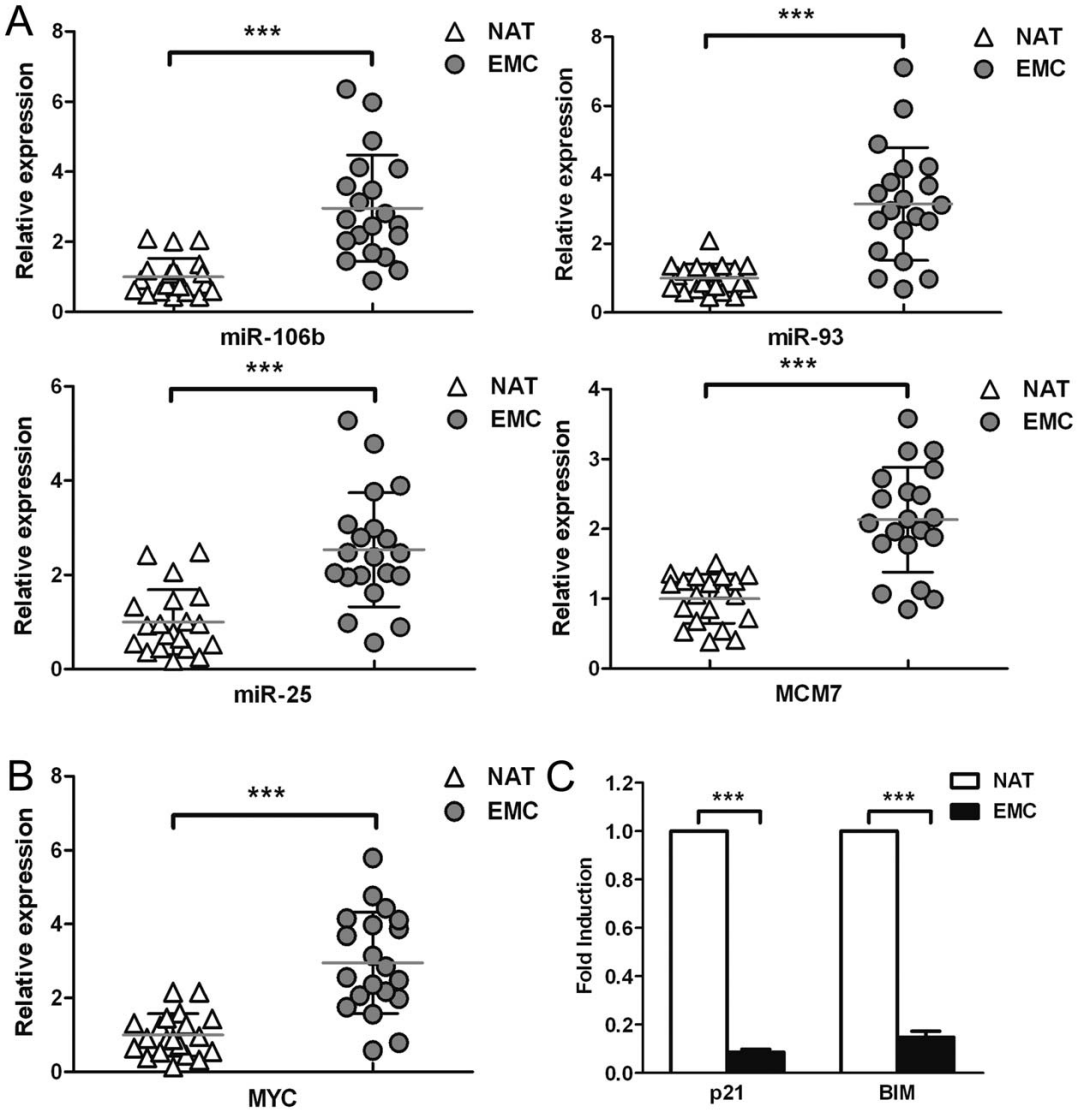
qRT-PCR analysis of miRNAs and related transcripts in clinical EMC tissues. (**a**) Twenty clinical EMC samples and the normal adjacent tissues (NAT) were analyzed. The average expression values of adjacent normal tissues is set at 1. Horizontal bars represent average levels. The expression of miR-106b, miR-93, miR-25 and their host gene MCM7 were upregulated in EMC tissues compared to the normal adjacent tissues. (**b**) MYC expression was upregulated in EMC tissues. (**c**) P21 and BIM expression in EMC tissues. ***, P<0.001, paired t-test.

We next analyzed expression of the *miR-106b-93-25* cluster and the host gene *MCM7* in clinical EMC samples. Our data showed that all three miRNAs, as well as *MCM7*, were upregulated by more than 2-fold in clinical EMC samples compared to the normal adjacent tissue (Fig. 6A). The expression profiles of *MCM7* mRNA and its resident *miR-106b-93-25* cluster showed a significant correlation between the host gene and intronic miRNA cluster expression. In addition, we also found that the expression of MYC was upregulated (Fig. 6B), while *p21* and *BIM* were downregulated in clinical EMC samples compared to the adjacent normal tissue (Fig. 6C).

## Discussion

HDAC inhibitors, having many effects on various types of cellular functions, can modulate the transcription of cancer-related gene expression by histone acetylation [25], [26] and thus be considered as candidate drugs in cancer therapy [27], [28]. As a well-known HDAC inhibitor, TSA inhibits all class I and II HDACs, and has potent anti-proliferation properties in a variety of cancer cell lines [29]. In recent years, its function in endometrial cancer cells has attracted more and more attention. Wu Y and Guo W found TSA suppresses proliferation of endometrial stromal cells through induction of cell cycle arrest and p21 [30]. The regulation of IL-1beta-induced COX-2 expression and TNFalpha-stimulated nuclear factor kappa B activation, as well as the ability to attenuate invasiveness by TSA may also contribute to its anti-proliferation and pro-apoptotic properties in these cells [31], [32], [33], [34]. Moreover, studies also showed a profound antigrowth activity of TSA in endometrial cancer cells, including anti-proliferation, cell cycle arrest and apoptosis [35], [36]. However, till now, the precise mechanism of TSA suppression of the malignant phenotype of EMC cells is still not fully elucidated. In this study, we showed that miRNA, a wide-spread novel transcriptional regulator, is involved and tightly regulated in the anti-tumor function of TSA in EMC cells, which is in consistent with clinical founding, and thus providing a new mechanism of TSA function in cancer cells.

The *miR-106b-93-25* cluster is composed of the highly conserved *miR-106b*, *miR-93*, and *miR-25* that have been shown to accumulate in different types of cancer, including gastric cancer [37], prostate cancer [38], and esophageal adenocarcinoma [39], hepatocellular carcinoma [40], and multiple myeloma [41]. *MCM7*, the host gene of *miR-106b-93-25* cluster, is a transcription factor that controls the G1-S transition, activating a variety of genes involved in DNA replication, and belongs to a family of specialized proteins that licenses chromosomal DNA to undergo replication once, and not more than once, at each cell cycle [42]. Higher expression level of *MCM7* is also observed in many cancers and regarded as an indicator of poor prognosis [43]. Thus the synergistic effect of *miR-106b-93-25* cluster and *MCM7* may account for the over-proliferation property of some tumor cells. In this study, we found TSA treatment significantly suppressed *miR-106b-93-25* cluster as well as its host gene *MCM7* in EMC cells (Fig. 1D). We further demonstrated that the reduction of *miR-106b-93-25* cluster and *MCM7* resulted from the down-regulation of *MYC* in TSA treated EMC cells. Actually, there is an E-box sequence in *MCM7* promoter and MYC can bind to this sequence to regulate *MCM7* transcription (Fig. 5A–D). Because *MCM7* mRNA and *miR-106b-93-25* precursors are co-transcribed, MYC was observed to regulate the expressions of both *MCM7* and *miR-106b-93-25*. In fact, MYC increased the expressions of *MCM7* and *miR-106b-93-25* with identical kinetics, whereas *MYC* silencing by siRNA paralleled the downregulation of *miR-106b-93-25* (Fig. 4B&4C). It is very reasonable that other *MCM7* transcriptional regulators, such as E2F1 can also activate this cluster in specific contexts [43], [44]. However, we have not found a clear correlation between E2F1 expression and *miR-106b-93-25* level in EMC cells (data not shown).

*p21* and *BIM* are target genes of *miR-106b* and *miR-25* respectively [18]. Our data confirmed this in EMC cells (Fig. 3A–C). Moreover, due to the suppression of *miR-106b-93-25* cluster in TSA treated EMC cells, p21 and BIM dramatically increased and eventually led to cell cycle arrest and apoptosis. p21 was reported to be up-regulated after TSA treatment in different cells by several independent groups [30], [45], [46]. The main explanation of this phenomenon lies in the up-regulation of p53. The expression level of p53 elevates in the presence of TSA, leading to the up-regulation of p21 [22]. Besides this p53-dependent pathway, recent studies have demonstrated that the HDAC inhibitor-mediated induction of *p21* is the result of increased H3 and H4 acetylation associated with the *p21* gene promoter [20], [21], [47]. However, our studies showed another mechanism of p21 up-regulation in TSA treated cells which is totally different from the previous ones (Fig. S2). We showed in our data that TSA triggers the suppression of *MYC* and its target gene *MCM7* and *miR-106b-93-25* cluster, resulting in the releasing inhibitory function of *miR-106b-93-25* cluster and up-regulation of p21 and BIM, which in turn induce cell cycle arrest and apoptosis in EMC cells (Fig. S2).

In summary, the current work revealed how TSA restricts EMC cells proliferation and regulates their apoptosis via miRNA. The work demonstrated the critical role for *miR-106b-93-25* cluster in this process, advancing our understanding of the mechanism of TSA anti-tumor function in EMC cells.

## Supporting Information

Figure S1 Efficiency of different MYC-siRNA. ECC-1 cells were transiently transfected with three siRNA vectors targeting different sequences of human *MYC*. The protein level of human MYC was detected by Western blot analysis. The MYC-siRNA3 was the most effective in silencing the MYC gene.
(TIF)

Figure S2 Schematic diagram of TSA inhibition of MYC and *miR-106b-93-25* cluster pathway of EMC oncogenesis.
(TIF)

Table S1 Primer sequences.
(DOC)
