# Supplementary figures and images for: TSA Suppresses miR-106b-93-25 Cluster Expression through Downregulation of MYC and Inhibits Proliferation and Induces Apoptosis in Human EMC
Source: PLoS One. 2012 Sep 19;7(9):e45133. doi: 10.1371/journal.pone.0045133 (PMC3446970; doi:10.1371/journal.pone.0045133)

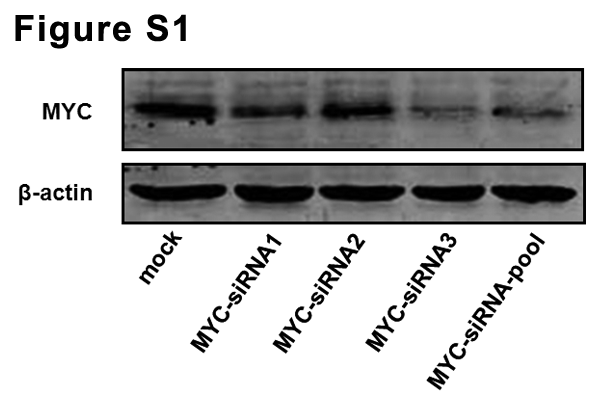

Supplement: Figure S1 — Efficiency of different MYC-siRNA. ECC-1 cells were transiently transfected with three siRNA vectors targeting different sequences of human MYC. The protein level of human MYC was detected by Western blot analysis. The MYC-siRNA3 was the most effective in silencing the MYC gene. (TIF) [file pone.0045133.s001.tif]

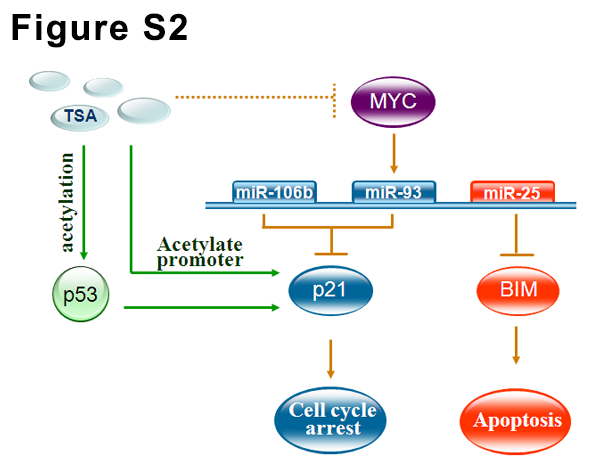

Supplement: Figure S2 — Schematic diagram of TSA inhibition of MYC and miR-106b-93-25 cluster pathway of EMC oncogenesis. (TIF) [file pone.0045133.s002.tif]
