# Supplementary material for: TSA Suppresses miR-106b-93-25 Cluster Expression through Downregulation of MYC and Inhibits Proliferation and Induces Apoptosis in Human EMC
Source: PLoS One. 2012 Sep 19;7(9):e45133. doi: 10.1371/journal.pone.0045133 (PMC3446970; doi:10.1371/journal.pone.0045133)
Supplement: Table S1 — Primer sequences. (DOC) [file pone.0045133.s003.doc]

**Supplemental Materials**

**Table S1 Primer sequences**

| **Primes** | **Sequences (5’-3’)** |
| --- | --- |
| has-miR-106b | TAAAGTGCTGACAGTGCAGAT |
| has-miR-93 | CAAAGTGCTGTTCGTGCAGGTAG |
| has-miR-25 | CATTGCACTTGTCTCGGTCTGA |
| qMYCF | CGTCTCCACACATCAGCACAA |
| qMYCR | CACTGTCCAACTTGACCCTCTTG |
| qMCM7F | GGGGCTCCAGATTCATCAAAT |
| qMCM7R | CAAGAAAATACCAGTGACGCTGAC |
| BIM_UTR_up | GAATTCGCTCCCAGTTAAGTAACTTGAC |
| BIM_UTR_dn | CTGCAGTTATTTACAGCAG |
| BIM mutant F | TGCCACAAAACTTGTGCAATAC |
| BIM mutant R | ACAAGTTTTGTGGCAATTACCC |
| p21_UTR_up | ACACTCAGACCTGAATTC |
| p21_UTR_dn | CTGCAGCCACCATCTTAAAATGTCTG |
| p21 mutant F | TTGCACAAAGATTAGCAGCGGAA |
| p21 mutant R | TAATCTTTGTGCAATGAACTG |
| qp21F | AGCAGAGGAAGACCATGTGGAC |
| qp21R | TTTCGACCCTGAGAGTCTCCAG |
| qBIMF | TGCAGACATTTTGCTTGTTCAA |
| qBIMR | GAACCGCTGGCTGCATAATAAT |
| MCM7_ChiP_F | CGTCACTCATTCTAGGCC |
| MCM7_ChiP_R | TAGCGCGTAGTCCTTCAG |
| MCM7_up(-756) | CTCGAGAATGAACCAGTCCTGGGA |
| MCM7_up(-570) | CTCGAGACTCAGTGAAGGATCCTG |
| MCM7_up(-500) | TTTCTCGAGCAGCCCCAAGGGTCTAGG |
| MCM7_up(-403) | CTCGAGGACAAGAAGACGGCGAAAGTCG |
| MCM7_up(-185) | CTCGAGTAGCGGGAGGTGAAGAAGG |
| MCM7_up(-70) | CTCGAGATTCTCAGCTTCCCCAG |
| MCM7_up(-52) | CTCGAGAGGAGCAAGACCTCTGAG |
| MCM7_dn(44) | AAGCTTCGTAGACCCGTACCCTTCTCTAGC |
| MYC siRNA1 | GGAACUAUGACCUCGACUATT |
|  | UAGUCGAGGUCAUAGUUCCTG |
| MYC siRNA2 | CGGUGCAGCCGUAUUUCUATT |
|  | UAGAAAUACGGCUGCACCGAG |
| MYC siRNA3 | CAAGGUAGUUAUCCUUAAATT |
|  | UUUAAGGAUAACUACCUUGGG |
